# Rethinking trade-offs in nature-based solutions from a multispecies justice perspective

**DOI:** 10.1038/s42949-025-00261-5

**Published:** 2025-08-18

**Authors:** Katinka Wijsman, Melissa Pineda-Pinto, Simo Sarkki, Charlotte Stijnen, Janneke den Dekker-Arlain, Christopher M. Raymond

**Affiliations:** 1 Department of Human Geography and Spatial Planning, Utrecht University, Utrecht, The Netherlands; 2 Melbourne Centre for Cities, Faculty of Architecture Building and Planning, The University of Melbourne, Melbourne, VIC Australia; 3 Cultural Anthropology Unit, University of Oulu, Oulu, Finland; 4 University of Erfurt, Erfurt, Germany; 5 Helsinki Institute of Sustainability Science, University of Helsinki, Helsinki, Finland; 6 Ecosystems and Environment Research Program, Faculty of Biological and Environmental Sciences, University of Helsinki, Helsinki, Finland; 7 Department of Economics and Management, Faculty of Agriculture and Forestry, University of Helsinki, Helsinki, Finland

**Keywords:** Environmental social sciences, Environmental studies

## Abstract

Trade-offs in nature-based solutions are increasingly recognized, with novel research interrogating their justice implications. Yet, these trade-offs and justice implications remain entrenched in an anthropocentric orientation, which is problematic in ecological and ethical terms. We discuss four common assumptions on trade-offs in NBS (instrumentalism, neutrality of science, collaborative consensus, and unitemporality) and rethink them through a multispecies justice lens, maintaining that dealing with trade-offs is a form of interspecies politics.

## Introduction

Research on the co-benefits of Nature Based Solutions (NBS) (e.g., refs. ^1,2^) typically assesses how diverse ecosystem services (e.g., carbon sequestration, pollination, air and water purification) and wider elements of health and livelihood (such as risk reduction, equity, sustainability transformation) can be simultaneously served through NBS (see ref. ^3^). Besides the potential for synergies between diverse ecosystem services, however, assessments also reveal disservices (e.g. refs. ^4,5^) and a potential for conflict between and among ecologically and socially desirable outcomes^6–10^. The consideration of potential trade-offs and their effects in practice reflects a recognition that multiple benefits can conflict with one another, and that choices are inherent in the design and functioning of NBS, with implications for their desirability and appropriateness. Yet, the justice implications of how trade-offs are discussed and settled remain largely overlooked^8^, despite justice having become a key concern in NBS applications^11^. Leaving justice considerations in the periphery can increase, entrench, and aggravate social-ecological inequalities and vulnerabilities, particularly for individuals and communities that are already at risk. If NBS are to be designed and implemented in just ways, trade-off thinking needs to make justice implications explicit.

At the same time that scholarship on trade-offs largely overlooks questions of justice, discussions on justice in NBS have traditionally proceeded from an anthropocentric viewpoint^12–14^. The discussion of the distribution of benefits and burdens, the participation in decision-making, and the recognition of stakes typically proceeds from the perspective and interests of human individuals and communities. However, given that choices in the design and implementation of NBS can have repercussions ranging from positive to detrimental for many living beings (and their relations) beyond the human, a narrow focus on humans is problematic in both ecological and ethical terms^15–17^. In ecological terms, a narrow focus on humans misses out on ecological relationships that are easily overlooked in an Anthropocentric frame and risks undermining ecological integrity. In ethical terms, failing to acknowledge the value (beyond human utility), capabilities, and needs of the wider living world is a moral mistake, undermining multispecies justice (MSJ). It is in this context of recognition and consideration that justice is reconceptualized, and the subject of justice expanded to include nonhuman living beings and the interlinkages between species through the notion of MSJ^16^. At its core, MSJ recognizes that nonhuman beings and ecosystems can be bearers of rights and deserving of equitable consideration in decision-making processes, and advocates ethical relationships with and responsibilities to these species and ecosystems^18,19^. It is therefore critical to consider MSJ as a process and practice of reflexivity, not only a set of outcomes to be examined^13^.

This article does some of this reflexive work. Taking MSJ as a starting point, in this article, we expand the notion of which actors are due consideration in thinking about synergies and trade-offs in NBS design and implementation. In particular, we rethink trade-offs in NBS, asking how the expansion of the notion of justice to a wider set of subjects changes the characteristics, conflicts, and concerns that we see in NBS trade-off assessments. We thus focus on trade-offs rather than co-benefits, acknowledging that MSJ can also bring synergies into view, but insisting on the crucial work of considering who “loses out” in those cases that synergetic win–win scenarios are not possible. We stress that, ultimately, the ethical crux of working with NBS lies in how trade-offs are considered and dealt with. We do the work of rethinking trade-offs by critically discussing four assumptions underlying trade-off thinking in NBS, either explicitly or implicitly: an assumption of instrumentality, one of neutrality of science, one of collaborative consensus, and one of unitemporality. Each of these assumptions is discussed by bringing NBS and MSJ literatures into conversation with each other, and illustrated through a case study of urban NBS. We propose alternative foci for NBS trade-off discussions informed by the MSJ lens, and conclude that trade-offs should be understood as an issue of inter- and intraspecies politics, where being explicit about the stakes and justice implications of NBS interventions from a more-than-human perspective is both ecologically and ethically necessary. MSJ does not endorse a simple solution to the management of complex trade-offs, but rather an alternative lens through which to identify challenges, as well as understand problems and conflicts.

## Ecocentric politics and ethics: lessons about multispecies spaces for multispecies justice

The human-centered approach to justice considerations in NBS has recently been challenged^12,14^. Central to this call to abandon anthropocentrism is the urge to consider benefits and harms related to NBS interventions from a multispecies perspective, including more-than-human beings and ecological communities' needs and interests. This expansive framing of multispecies benefits and harms is at the core of MSJ: an ethical and political project that argues for the moral consideration of nonhuman beings as bearers of justice^16^. MSJ is both an ethical and a political project because it fundamentally re-evaluates our moral obligations to the nonhuman world, and seeks to translate these obligations into concrete changes in our social, legal, and political structures. Ethically founded on the idea of intrinsic value, the understanding that “all living organisms have a good of their own”^20^ and “that nonhuman beings are not simply of value as a means to an end”^21^, the crux of this thinking is that beings have value independent of human interests. The ethical project of MSJ argues that intrinsic value warrants extending moral consideration, responsibility for, and respect to all living beings^16,22^. Politically, this framework necessitates a rethinking of justice itself, aiming to broaden its scope beyond humans to include diverse species and their relationships, ultimately advocating for new forms of governance, laws, and social practices that foster a more just and sustainable multispecies world^23^.

Fundamental to this ethico-political project is MSJ recognition of the agency and capabilities of living beings—that is, their functionings and abilities to flourish, develop, and live a life worth living^16,24–26^. For some scholars, this is justified through arguments of animal sentience^25^, whilst others argue for the integrity of life^27–29^. These arguments underline the interdependence and interconnectedness between living beings (including humans) and ecosystems, and maintain that extending moral consideration to the more-than-human realm opens diverse ways to include multispecies needs and interests in decision-making processes. This can be expressed by being more attentive, responsive, and embracing of more-than-human beings and collectives, as well as ways of knowing and being. Going further, rights-based approaches call for deeper political and legal transformations that challenge colonial, destructive, and violent legacies and histories^30^. This can lead to rethinking political processes and institutions that support reparations and Indigenous self-determination. It is important to recognize that MSJ aligns with and builds on Indigenous and relational worldviews, and similar ethical frameworks like the ethics of care, that emphasize deep, reciprocal relationships between humans and the natural world, viewing land, waters, animals, and plants as kin or relatives. Ethics are rooted in responsibility, reciprocity, and interconnectedness, rather than individual rights or instrumental value. When encountering conflicts and trade-offs, these frameworks, like MSJ, seek solutions that maintain the health of relationships, often prioritizing long-term ecological balance and bio-cultural continuity over short-term human gain.

The extension of moral consideration, however, does not by itself provide a balanced and conflict-free situation. On the contrary, it highlights and obliges us to think about the many and complex effects of decisions, actions, and types of relations that unfold across human and more-than-human worlds, the stakes of these decisions, and the processes through which we organize them. MSJ does not offer a set of normative principles or a universalist approach that can be implemented in different contexts and settings. While this could be considered a shortcoming in the eyes of those thinking about applications of MSJ, what MSJ offers is a starting point to recognize conflict and trade-offs through place-based approaches. MSJ does not eliminate conflict; it provides a framework for acknowledging the full spectrum of beings involved, understanding their interconnectedness, and seeking solutions that do not simply prioritize human interests. Put differently, the extension of moral consideration begs the question of interspecies politics that is attentive to the relations and interactions between multiple species “about the conditions of shared life”^31^.

Drawing attention to several ethical dilemmas, it requires us to ask: what are the obligations that MSJ imposes? For example, when can wild animals or introduced plants be treated as disruptive in urban settings and rendered removable or killable if they also deserve moral consideration? How do we recognize and deal with conflicts of interest between different more-than-human species^32,33^ or between more-than-human species and disadvantaged communities^32,34,35^? When do humans have an obligation of action, and when is human interference a problem? How should we tackle a lack of human willingness to coexist with other species, whether or not based on human supremacist thought? And how do we change the techniques, ideas, and terminology that constitute a logic of domination? In short, MSJ insists on attention to the intra- and interspecies politics and ethics of environmental beings and communities that seek an “inclusive, relational ecological world as the shared community of justice”.

At the same time that an MSJ approach underlines conflicts and trade-offs by centering more-than-human beings and communities, the approach is powerful because it does not exclude humans from consideration. Instead, by emphasizing interconnectedness and mutual dependence, MSJ draws attention to shared vulnerabilities and resilience. For example, authors writing from an MSJ perspective have addressed entrenched dehumanization while acknowledging the real, embodied hardship of social inequalities, multidimensional poverty, and pervasive injustices in all societies’^16^, and asked whether the institutionalization of rights to nature is a continuation of Western, colonial tools of oppression^36^. What MSJ offers is a radically different way to debate, think about, and transform socio-natural and political processes.

## Interrogating stubborn NBS trade-off assumptions

For urban NBS, a crucial question that emerges from the moral consideration of more-than-human others is how the concept of MSJ governs decisions about what NBS should look like, what living beings should be helped in their survival through NBS, and how these decisions should be made. In this section, we turn to this question from the vantage point of trade-offs in the design, planning, implementation, and use phases of urban NBS. As situations where achieving one thing requires giving up another, trade-offs are where conflicts and priorities come into play, and where decision-making involves making choices between competing alternatives with consequences for who receive the benefits or carry the costs of NBS interventions. As such, trade-offs in NBS can be considered political^8^, but “allegories of good versus evil” are unhelpful in understanding and dealing with trade-offs because they are often complex^37^. Yet, trade-offs are how competing visions of NBS are debated, and their settling is consequential in ecological, ethical, and political registers. Given this, below we interrogate four common assumptions in NBS trade-offs, informed by an MSJ perspective.

We examine four key stubborn assumptions on NBS design and implementation with respect to i) instrumentalism, ii) neutrality of science, iii) collaborative consensus, and iv) unitemporality. The assumption of instrumentalism regards the goals of NBS, which are typically defined from an anthropocentric viewpoint and treat ecological beings and processes as means towards ends. The assumption of neutrality of science concerns the knowledge base of NBS, which typically treats science as factual and outside of value judgment. The assumption of collaborative consensus regards the process of NBS, which prioritizes deliberative strategies that presume agreement can be achieved and conflict can be resolved. Finally, the assumption of unitemporality concerns the temporal scale of NBS, which remains entrenched in modern temporality. We identified these assumptions based on prior review work on trade-offs in NBS that highlights foci on ecosystem services, land-use conflict, governance values, and socio-ecological dynamics^8^. Based on this systematic review, we went through a brainstorm and grouping exercise, through which it became evident that these assumptions are among the most widely held assumptions in NBS policy and governance, and that these assumptions show to be problematic once we take MSJ as the central ambition in the application of NBS. The assumptions and the implications of their problematization through an MSJ lens are illustrated with short case study vignettes. These case studies represent different life forms (rivers, plants, birds, insects; all in relation to other species) and are taken from various regions (Central America, North America, Europe, and Oceania), to showcase how questioning these stubborn assumptions is of relevance in a variety of different settings.

### Assumption of instrumentalism

It is common practice in NBS planning to understand trade-offs by focusing on ecosystem services and disservices (ES(D)) and mapping how these interact or conflict, in theory (during the NBS design phase) and in practice (once NBS are implemented) (e.g., refs. 38–40) The framework of ES(D) is a tool to understand which services, understood as benefits, NBS are able to provide at the cost of others, while enabling a translation of generic stakeholder interests and preferences into explicit terms. Thereby, the ES framework can map how different stakeholders' preferences can find synergies or stand in conflict with each other. For example, Mosleh et al.^41^ indicate the differences in preferred ES for green infrastructure planning at the city level, while Wagner et al.^42^ illustrate the potential discrepancies between the desired ES of an area and the (in)ability of the area to provide those services. The distribution of benefits (services) such as recreational opportunities, esthetic value, and the provision of shade; and negative impacts (disservices) such as allergenicity, unwanted species, and perceived infrastructure risks is central herein^43–45^. A focus on stakeholder perceptions, preferences, and participation in NBS trade-off analyses^9,46^ seeks to bring a focus on inclusivity and justice of NBS processes and outcomes.

Although ecosystem services and disservices provide a useful framework for understanding trade-offs and conflicts during the design, planning, implementation, and use phases of NBS, it has ethical and practical limitations. Ethically, the ES(D) framework inherently values nature based on the services it provides to humans, often emphasizing those that can be easily quantified or have direct economic benefits (e.g., water purification, carbon sequestration, flood regulation), whilst undervaluing the intrinsic worth of biodiversity, ecological communities and ecological processes^47^. This can contribute to the commodification of nature and, in turn, allow for market-based mechanisms (e.g., carbon markets, biodiversity offsets) where nature is treated as a form of capital that can be traded or offset^48^ and has been an ineffective tool in achieving public support for nature conservation^49^. At a practical level, the predominance of a single-city and single-service focus prevents a holistic assessment of biodiversity and ecosystem services synergies and trade-offs^50^, which is amplified by a misunderstanding between ecosystem functions and biodiversity^51^. These analyses reveal that “[t]he impact of trade-offs is often related to the intensity of use of ecosystem[s]” and that the most frequent trade-offs occur among provisioning and regulating ES^46^. An MSJ lens invites researchers and planners to consider how exactly life-supporting ecosystem functions at the foundation of human and non-human species’ wellbeing are negatively impacted. For example, how the misappropriation and biopiracy of Indigenous ecological knowledge affects the stewardship of ecosystems, and the well-being of Indigenous communities^52^, or how discounting biodiversity and ecological communities that do not hold a significant value for people can risk the ecological integrity of nature-based solutions^17^.

A MSJ perspective also invites a deeper understanding of who benefits, who bears the harms, who is recognized and who is counted through NBS planning (see Case 1). Broader framings and deeper analyses that recognize the interconnections and relationality of the complex interrelated multispecies world we inhabit are now considered essential^51,53^. Similar arguments have been raised regarding the plural valuation of nature as a way to foster inclusivity amongst “benefits, stakeholders, knowledge systems and worldviews”^54–56^, and as a means to foster recognitional justice through the diverse representation and empowerment of marginalized worldviews^57^. Such a recognition requires moving away from a perspective of instrumental value, in which nature is valued as a means to satisfy human needs and interests and which still dominates ES literature^58^, and instead incorporates non-instrumental and plural values.

#### Case 1: Interurban Biological Corridors, San José, Costa Rica

The Interurban Biological Corridors is a nature-based planning initiative in the metropolitan urban area of San José, Costa Rica. The corridors are developed as mechanisms for ecological conservation and social-ecological wellbeing^59^ by concretizing a vision of social-ecological connectivity, restoration, and conservation in cities. The interurban biological corridors connect protected areas at the regional scale, promoting habitat restoration, wildlife movement, and preservation of genetic diversity through the protection of river basins^60^. These Corridors, however, are also framed and managed as a social-ecological tool to improve human wellbeing; its effectiveness is measured and management interventions are based on the ES(D) framework^61^. To understand trade-offs, the managing institution uses an “analysis of conflict zones” approach to understand how, why, and between whom conflicts emerge and play out. This framework locates the emerging and unfolding of conflict in land-use practices, people’s values, tensions between biodiversity conservation and establishing protection areas, and tensions due to land tenure and the use of the river as a resource. Trade-offs and conflicts are managed through a zoning approach aiming to regulate land-use changes in a top-down fashion, and based on principles of productivity and efficiency of space. While thus managing certain trade-offs, it simultaneously generates new trade-offs, such as polluted and environmentally degraded areas, areas that are protected in a static way, and unevenly distributed burdens and benefits^62^.

Bringing the MSJ approach to bear on the Corridors, a different picture emerges. Rivers would be considered a living system, rather than a resource for consumption or a production point of services. This could be recognized through the provision of legal personhood and independent rights to flourish; or through Indigenous collaborative governance representing the river’s stakes in decision making settings^63^ “Management” then becomes undesirable, as that would misrecognize the “radically diverse life project, capabilities, phenomenologies, ways of being, functionings, forms of integrity, and relationalities”^16^ of living organisms. Instead, the recognition and dealing with trade-offs and conflict would have to facilitate a process that considers the interest of the river, *as a living being*, rather than the interests of those around the river, as a resource. For example, diverting water for irrigation is not just a technical or economic decision—it becomes a question of whether such an action infringes upon the river’s “right” or capabilities to flow, to sustain its ecosystems, or to exist in a healthy state. This reframes trade-offs as negotiations among stakeholders, where the river’s needs—such as maintaining ecological integrity, flow, and self-renewal—hold similar weight to human interests.

This shift complicates trade-offs, making them less about optimizing outcomes and more about navigating responsibilities, obligations, and relationships. It may lead to decisions that favor ecological integrity over short-term economic gain, or that prioritize restoration over exploitation. Ultimately, including a river in justice considerations invites a broader ethical imagination: one in which trade-offs are not simply technical dilemmas but moral dialogs among interdependent beings. It challenges dominant anthropocentric paradigms and opens space for more reciprocal, respectful, and sustainable ways of relating to the more-than-human world.

### Assumption of neutrality of science

A core feature of NBS is that they should provide environmental benefits alongside social and economic ones, contributing to biodiverse futures and healthy ecosystems simultaneously. Knowledge stemming from the subdisciplines of ecology is therefore seen as pivotal to guide the planning and management of NBS, and to understand potential trade-offs between management options to ultimately ensure positive contributions to biodiversity. This knowledge comes into play, for example, through the provision of indicators, which are thought to capture potential improvement or deterioration of biodiversity through NBS by looking at such things as species variation and diversity, and by sorting native from non-native species (e.g. refs. ^64–66^. For example, assessments of trade-offs related to biodiversity place a strict division between native and non-invasive species as being conducive to biodiversity, whereas non-natives and invasives are seen as disservice to biodiversity^64^. The underlying assumption is that ecological knowledge can objectively bring into view the benefits to biodiversity that NBS provide, and that the ecological concepts and techniques used facilitate an impartial description unhindered by bias.

While ecological knowledge on trade-offs brings much needed attention to issues of environmental processes and quality, and to the multifariousness of (managing for) ecological benefits (e.g. ref. ^67^), important ethical and political dimensions regarding the stakes for and treatment of diverse living beings in NBS have been overlooked due to the ways we conceptualize and represent relationships between and among different species. These dimensions come into view when we broaden the scope of consideration in NBS as inspired by an MSJ lens, challenging narrow or omitted representation of diverse species in NBS^13^. Here, we argue that this representation is not merely of concern regarding planning discourses but equally involves reflexive consideration of the conceptual and epistemological apparatus of science. *How* we know affects *what* we know, and ongoing engagement with concepts, frameworks, and practices is therefore critical to reveal blind spots and offer alternative approaches to understanding trade-offs in NBS in ways that are more conducive to MSJ^68–70^.

Two developments in ecological science illustrate the need to trouble the common assumption of neutrality of ecological knowledge and the indicators for NBS derivative of it: the widespread disregard of the cognitive and emotional capacities of animals, and their well-being; and the insistence on nativeness as a conservation principle. As our understanding deepens regarding the cognitive and emotional capacities of animals—and even the abilities of plants—our approach to ecological knowledge is undergoing a profound transformation. No longer can we view ecosystems as collections of passive resources or lifeforms as mere data points. Instead, we are being called to recognize them as active participants in their own right, capable of experience, response, and relationship. For example, animal intelligence has long been disregarded in ecological scientific practice, where behaviorist views of animals as stimulus-response machines were dominant. This disregard is a problem when we consider that the capacity for intelligence is a prominent basis upon which moral consideration is dispensed, and the Descartian model of the animal as automaton popular in science ipso facto excludes animals from moral consideration. Recent work on animal cognition challenges this view of animals, reminding us that our scientific concepts and models are not outside of scrutiny, and that reflection on their limits remains important, especially when those scientific categorizations and understandings carry ethical and political implications of worth and consideration^65^. The exclusion of a concern for animal well-being in ecological sciences based on a supposed incommensurability with processes of predation and death, then can be called into question as well, based on the duty of moral consideration (whether based on intelligence, sentience, or another criterium). Similarly, typical classificatory systems of ecology, that sort out belonging and desirability of plant and animal species by differentiating between native and non-native species, can be questioned from an MSJ perspective^13,71^. Sometimes non-native species are harmless, provide benefits to their environments, and have conservation value^72–74^. More fundamentally, species travel, landscapes are dynamic, and many ecosystems are transformed by humans, making the designation “native” to some extent arbitrary and linked to a Romantic notion of pristine Nature^75,76^. The common conceptualization of native species as good and non-native species as bad, typically accompanied with metaphors drawing action into militaristic terms of fighting^77^, might not serve conservation efforts in the way that is customarily thought^74^^,^^78^. The presumed neutrality of science might then get in the way of alternative frames, such as cohabitation^79^.

Taking a MSJ perspective, which brings attention to the ethical and political dimensions of species and their organization, the critique of the assumption of neutrality is that ecological knowledge—like all knowledge—is not purely objective or universal, but instead influenced by broader social, cultural, and historical contexts, so that it becomes governed by discursive framings that inadvertently express ontological views and value judgment^80,81^. Paying attention to such framings and how they enable or hinder justice, then, is a key analytical task^71^. From the MSJ perspective we can see that, for instance, once animals are rendered intelligent, or animal sentience and welfare become a legitimate concern, the design and maintenance of NBS should not merely focus on the management of specific species, but should take life trajectories into consideration that acknowledge that NBS and the ecosystems making them up are multispecies communities in which social bonds are key^71,79^. Taking such a relational understanding of moral significance could thus enable NBS scholars to rethink the “entities” traded off, as not merely about ecosystem services, but different interspecies constellations, thus recognizing sentience and sociability as dimensions impacting the justness of decisions. Not doing so risks failure to account for the needs and well-being from an intrinsic point of view and thus potential exploitation or mistreatment. Furthermore, an MSJ perspective invites us to have a more contextual understanding of the threats posed by non-native species. This includes rethinking which trade-offs are captured, and how to recategorise the threats and benefits posed by non-natives based on their combined ecological and human wellbeing benefits or harms (Case 2). Overall, the lessons from MSJ invite us to take a second look at the ecological concepts and knowledge we rely on in our approach to NBS, acknowledging that these concepts and knowledge have histories, alternatives, and world-creating abilities. They are not politically or morally innocent, but instead influence academic and policy practice in ways that can stand in tension to goals of justice. To move beyond the assumption of the neutrality of science, then, means to practice reflexivity to enable forms of accountable and just practices.

#### Case 2: Urban wetlands and Phragmites Australis in North East America

Land managers of urban wetlands focus their efforts on limiting the dangers to the health of these ecosystems, in order for them to provide habitat and food for a biodiverse constituency and to offer ecosystem services to humans as well. The control of invasive species, for example through removal projects, is a key management strategy for wetland health. In the American North East, the *Phragmites Australis*, or common reed, is one of the main targets to control or eradicate, and lots of time, effort, and money are spent on removal. The non-native strain of this aquatic grass, introduced some 200 years ago through ship ballast, is now the most common Phragmites on the East Coast. It is typically seen as harmful because it is believed to compete with native vegetation, crowding or shading it out, and to alter habitats in ways that are inhospitable to diverse bird, fish, insect, and invertebrate species, destroying sites for reproduction and rearing of the young.

And yet, the onset of climate change leads some ecologists and communities to reconsider the view of Phragmites as a pest and a danger to wildlife and plants. With sea levels rising and extreme weather events such as storm surges expected to become more common, urban wetlands are increasingly recognized and valued for their capacity to slow down wind and water and thus protect property and life. However, crunched by the speed of sea level rise and the inability to migrate inland because of human structures, some urban wetlands are at risk of drowning^82^. The deep roots and fast-growing, dense stands of stems of the Phragmites, its ability to stabilize soil, and its ability to create more detritus and opportunity for elevation than other grass species, “may provide resource managers with a strategy of combating sea-level rise”^83^. Added benefits in the context of climate change are its ability to sequester carbon dioxide^84^ and nitrogen^85^. Killing and removing Phragmites may make soils, especially on vulnerable coastlines, more prone to erosion.

The reconsideration of Phragmites’ bad reputation should, however, not only be understood in terms of trading off biodiversity concerns over climate change impact considerations. The common sense knowledge on invasive species as harmful by definition has been questioned, with research suggesting that assumptions about low habitat value, detriment to biodiversity, and disturbance of marsh function may not hold in each case^86–89^. What is more, the eradication of Phragmites does not “just” kill these grasses, but impacts other species—including the native muskrat, American beaver, cottontail and diverse insects—who eat and live in these plants^87^. This suggests that specificity matters, and that the treatment of native and invasive species should be context-specific, especially in contexts like the urban, where environmental altercation has been irreversible and long-lasting.

### Assumption of collaborative consensus

Recent literature emphasizes the need for collaborative approaches in NBS planning and implementation through concepts like co-creation^90,91^, co-production^92,93^, co-design^94^, and collaborative governance^95,96^. These approaches are thought to help manage trade-offs and catalyze synergies in NBS implementation^97^, assuming that consensus between diverse actors can be found^98^. For example, it has been argued that searching consensus on the benefits and costs of NBS can help decision-makers to design and implement NBS^4,99,100^, and accounting for stakeholders’ perceptions on the likely co-benefits can enhance acceptability of the NBS^9^. However, these views lack sensitivity towards diverse perspectives on the claimed co-benefits; in particular, multispecies perspectives are generally lacking (exceptions refs. ^14,101^).

The assumption of co-benefits is debatable when we take an MSJ perspective, because it challenges the subject of benefit, recognizing that the distribution of harms depends on how the relationships between humans and other species are considered within specific contexts. For example, the habitats provided by NBS can also attract wild animals that are unwanted by their human neighbors, while rewilding efforts can be at odds with creating culturally appropriate foodscapes^34^. One way to manage multispecies conflicts is to include more-than-human species in an “interspecies democracy” by considering their actions as a form of political communication^31,102^. Furthermore, more-than-human species are also embedded in historical and political contexts, which shape multispecies coexistence and conflicts^103^. Assuming consensual co-benefits of NBS often masks legitimately diverse viewpoints behind the alleged consensus^104^. Theories of rational deliberation have been criticized for their ignorance of conflict and the benefits of conflicts for democratic societies^105–107^. For example, the taken-for-grantedness of the idea that birds in urban green space will enhance human well-being^108,109^, may, in actuality, mask diverse conflicting views behind the assumed consensus on the co-benefits.

Overall, instead of assuming consensus on the co-benefits of NBS, we should interrogate whether and why consensus or dissensus is at play. Here, we should seek to understand the political and historical contexts of the multispecies encounters, the type of conflict occurring, the alternative ways to build consensus for conflict management, and how to engage more-than-human species in conflict management. Raymond et al. encourage the creation of “transdisciplinary safe” spaces for dealing with intractable conflicts, which are never complete in NBS planning when representing multispecies needs^13^.

#### Case 3: The conflict between barnacle geese and humans in Helsinki, Finland

This case illustrates the difficulty of consensus-seeking methods in managing conflicts, and MSJ can offer an alternative means of negotiating partial settlements. In the 1970s, barnacle geese were close to extinction and therefore protected under the Bern convention, while the hunting of these geese was forbidden through the EU’s Birds directive in 1979^110^. This led to revitalization of the population reaching 1.4 million in 2020^110^, with around 6000 nesting individuals since in Helsinki 2015^111^. The geese arrive in Finland during their spring migration, and stay there until winter retreats from their Arctic breeding grounds^112^.

The presence and litter of barnacle geese has been considered to decrease the attractiveness of urban green spaces for people, and makes cleaning of the parks more expensive and difficult^112^. In the media, arguments made range from those arguing that the protection of barnacle geese should be ended^113^, to those that state “goose rage” is incomprehensible^114^, and others that insist urgent solutions to a catastrophic geese problem are needed^115^. In 2024, the City of Helsinki launched a competition seeking new tools to collect litter from the city parks and beaches, and hired 45 employees because of the geese situation. It is suggested that a previously vibrant picnic culture has ended in the Kaivopuisto city park, nearby kindergartens do not make trips to the park anymore, and that prior to outdoor gymnastic sessions, the geese litter has to be collected in bags^116,117^.

Failed consensus-seeking management efforts include proposals for a branding campaign to produce a positive image of the geese, and geese guides, who would circle around the parks and tell people about the lives and positive aspects of the geese^118^. However, these proposals were not transformed into mainstream practices. Recently, the coexistence of geese and people has been proposed in urban green space management^111^, for example, by letting the city lawns grow higher to attract less geese, and by building small fences to limit the movements of baby geese who cannot fly^119,120^. From this checkered history with geese, what seems to be emerging are multispecies negotiations. In these negotiations, material changes in the physical environment (e.g., fences, dogs, height of the grass) enable changes in geese behavior in ways that support the subjective well-being of humans in urban areas.

### Assumption of unitemporality

Commonly, trade-off considerations in NBS focus on how different benefits and burdens manifest across time, from current situations to future scenarios^121,122^. For example, a study on the perceived benefits from a protected area in Manila, the Philippines, identifies current conflicting stakeholder interests between values for biodiversity, ecotourism, and urban development potential and the implications for future land-use changes^121^. Similarly, a study on conflicting values connected to urban coastal mangroves positions values in the present with projections for the future^123^. Some studies investigate changes over time, looking at the recent past, for example, tracing the dispersion of green spaces in a context of increasing urban development^124,125^, or investigating forced displacement in informal settlements resulting from greenbelt development^126^.

Yet such studies do not fully engage with the multiple temporalities of place. For example, historical time can be transformed by atmospheric carbon, reproductive time can be queered by microplastics, and species extinction affects notions of multispecies time (see ref. ^127^). In particular, studies commonly ignore the potential for phenological mismatches (e.g., between plants and pollinators), as well as mismatches between ecological, social, and political systems, for example the disconnects between short-term thinking of political cycles and the requirement for longer-term thinking to manage the biodiversity and climate crises (e.g., refs. ^127–129^) (Case 3). Also, benefits and burdens are not always expressed in the same time period^130–132^, and the *longue duree* has a lasting legacy on socio-ecological constellations and their perceived justice^19,133^. The MSJ lens shows that social life cannot be conceived as distinct from the environment, and therefore, a rapprochement of different temporal worlds is critical to addressing temporal mismatches. It requires an improved understanding of how time is both embedded and contested within the intertwined social and ecological crises^127^, and in socio-ecological practices. It requires a deeper exploration of lived, situated experiences that take shape in particular expressions of time^134^, including seasonal rhythms and the different cycles, durations, and tempos involved in specific nature management practices (*ibid*), as reflected in the case of urban beekeeping (Case 4). The MSJ lens thus encourages temporal reflection for NBS trade-offs: whose future do we care for? Whose pasts and presents do we recognize? How can we become attentive to a diversity of rhythms, life cycles, and experiences over species boundaries? ^76^

Two related analytical moves can help in attending to polytemporality^135^ and its repercussions for NBS trade-offs: decolonial thinking and more-than-human thinking. First, decolonial thinking reveals how colonial narratives of “Nature” and practices of taming “Nature” endure in and influence NBS planning and governance^79,136,137^. For example, the siting of port cities and the design of drainage systems in Suriname and Guyana hinged on Dutch and British colonial ideas of waterways as controllable and exploitable, ideas that remain central through notions such as “flood control” and “utilizing natural resources”^79,138^. Even when biocentrist pre-colonial notions of “Nature” (as not separate from humanity, as something to be respected and lived in harmony with) are expressly revitalized in contemporary times, such as through Buen Vivir principles in Ecuador, this remains a theoretical exercise more than a success in practice^35^. Attending to this *longue duree* is a necessary step to understand how legacies of past social and multispecies injustice leave imprints on the present that may constrain breaking out of problematic pathways^79^. At the same time, scrutinizing colonial modes of planning and governance in NBS is a necessary step to recognize and redress past harms, current needs, and desired futures^136^. Second, more-than-human thinking challenges humanistic conceptions of time in favor of more-than-human timescales, assigning us to recalibrate ourselves to the different speeds of different forms of life^139^. This move brings into view how human timescales can disrupt those of other species, for example, when practices of environmental offsetting fail to account for the slowness of tree growth when a habitat sacrificed for urban development goals is supposedly unproblematically replaced by a new one^140^.

#### Case 4: Multispecies temporalities in urban beekeeping in Australia

Integrating beekeeping programs into urban areas is an NBS that serves as a refuge for pollinators^141^ and that fosters learning about biodiversity and responsible actions around the home or office^142^. Such programs can be used to address trade-offs, such as how the use of commercial bug-sprays (i.e., pesticides) can have sub-lethal effects on urban bee populations^143^. This brings into focus the plurality of times at play in beekeeping practices, where plural versions of the future (e.g., collapse, recovery) find expression in trade-offs in the present (e.g., harvesting for honey or leaving honey for bees; whether or not to manage swarms). Urban beekeeping, then, requires coordination between human and bee temporalities made through diverse and imbricated more-than-human relations^134,144,145^. Accounts of urban beekeeping inf Australia^134^ highlight that beekeeping requires a kind of “slowing down” and becoming “in tune” with bees—for example, slowing one’s body, focusing on bees, and keeping one’s distance.

The importance of time keeping and time telling over species boundaries is critical in beekeeping practices. Harvesting honey in ways that best protect the health of bees requires skillful sequencing, timing, and pacing of checking hives and the coordination of humans and bee tellings of time, which is never fully complete^134^. Beekeepers need to be attuned to the rhythms of seasonal changes, growth patterns, and circadian cycles to ensure the persistence of colonies. For example, beekeepers need to pay attention to the seasonality of the flowering of plants for bees (including differences in flowering frequencies, durations, and intensities between species), and the timing of swarming. Importantly, these plural temporalities are place-specific, meaning that urban beekeeping and its rhythms are not a homogenous (temporal) experience, but instead a situated practice^146^. In Australia, for example, where mass bee loss has not occurred and yet shapes attitudes towards beekeeping, honey production hinges on the irregular blossoming of Eucalyptus instead of seasonal cycles. The temporal coordination between diverse species that is critical to beekeeping is also diverse, warranting attention to the poly-temporal nature of the world. Such situated accounts of more-than-human relationships show how they stretch beyond current spacetimes (building on refs. ^134,147^), and that beekeeping is a negotiated, place-based practice where multiple more-than-human temporalities become entwined.

## Conclusion: necessary shifts for multispecies justice in trade-offs thinking for NBS

In this paper, we explored how the expansion of the notion of justice to a wider set of subjects changes the characteristics, conflicts, and concerns that we see in NBS trade-off assessments. Using insights from the project of MSJ, which urges us to attend to life projects and relationalities of living beings and systems for ecological and ethical reasons, we interrogated four common and unquestioned assumptions in how trade-offs are typically understood in NBS–assumptions of instrumentalism, neutrality, collaborative consensus, and unitemporality. MSJ helps us to rethink trade-offs and conflict in NBS by insisting on (1) plural and non-instrumental values; (2) alternative frames of knowledge production and assessment; (3) ongoing negotiations with political stakes; and (4) different temporalities coexisting and clashing. The resulting shifting terrain of approaching trade-offs (Table 1) therefore insists on the situatedness of NBS in political, ethical, and historical contexts alongside ecological ones, while questioning the usefulness of thinking about trade-offs in near-universal terms. Thus, theorizing trade-offs as an issue of intra- and interspecies politics, we argue for an approach to NBS trade-offs that is explicit about both the stakes and justice implications of NBS interventions beyond the human, and about the inevitability of making, sometimes difficult, choices.

**Table 1:** Traditional and MSJ-informed approaches to trade-offs in NBS

Assumption	Traditional approaches to trade-off assessments	MSJ perspectives on trade-offs
Instrumentalism	Focus on benefits/services and disservices	Recognition of plural and non-instrumental values and considers questions of costs and benefits “for whom?”, leading to more nuanced assessments of conflict.
Neutrality	Tendency to use indicators as measures of costs, benefits, and effectiveness, which are assumed to “objectively” measure reality	Emphasizes the ethical and political dimensions of costs and benefits, including issues of intelligence and sentience. Openness to critique assumptions of neutrality and reassess conservation principles.
Collaborative consensus	Assumes that consensus can be found by comparing and deliberating on co-benefits and costs within specific cases	Recognizes that consensus is unlikely. Rather, parties need to commit to an ongoing process of negotiation between human and more-than-human interests. The process needs to respect the situatedness of co-benefits and costs, including how injustices are embedded in historical and political contexts.
Unitemporality	Often views time in a single dimension, for example, as calendric or geological time.	Benefits and burdens are not always related to one time period or temporality of place. MSJ emphasizes a rapprochement of different temporal worlds, including how experiences are situated in different cycles, durations, and tempos over species boundaries.

Overall, the MSJ approach to NBS trade-offs challenges narratives of control and exploitation by centering more-than-human ontologies and epistemologies. How can we integrate such considerations into NBS planning and governance practice? A first step is to acknowledge that existing tools for trade-off assessments may be overlooking benefits and costs associated with NBS across different subjectivities. We therefore posit that the development of new trade-off tools, processes, and practices that attend to multispecies needs is warranted. What is more, it would be useful to have processes in place where constructive critique of the neutrality of trade-off assessment tool can be expressed, and find approaches through which to interrogate whether, how, and through what means collaborative consensus can be achieved—or how to make choices, and “pick sides”, in accountable ways, when conflict is inevitable. This means being more reflexive about what is being traded off and how it affects the well-being of humans and more-than-human species. Relatedly, planners need to respect the different temporalities of place and how this provides meanings and functions to both humans and more-than-human species, changing the way that trade-offs are recognized and distributed over space and time. In this regard, intergenerational thinking^148^ could become part and parcel of NBS trade-off assessments. All this together points to the need to find ways to fairly represent more-than-human perspectives—in both ecological and social terms—in planning processes in order to get a better understanding of the unfolding dynamics of interspecies relations in place and how these help—or hinder—some lives to flourish.

## Data Availability

No datasets were generated or analysed during the current study.
